# Monitoring Atypical Metabolite Biomarkers in Patients with Bile Acid Synthesis Disorders by a Novel Targeted Tandem Mass Spectrometry Assay

**DOI:** 10.3390/metabo16070436

**Published:** 2026-06-23

**Authors:** Kenneth D. R. Setchell, Xueheng Zhao, Stacey Reed, Wujuan Zhang

**Affiliations:** 1 Clinical Mass Spectrometry, Division of Pathology and Laboratory Medicine, Cincinnati Children’s Hospital Medical Center, Cincinnati, OH 45229, USA; xueheng.zhao@cchmc.org (X.Z.);; 2 Department of Pediatrics, University of Cincinnati College of Medicine, Cincinnati, OH 45227, USA

**Keywords:** bile acid synthesis disorders, atypical metabolites, biomarkers tandem mass spectrometry

## Abstract

**Background/Objectives**: Bile acid synthesis disorders (BASDs) represent a distinct category of progressive familiar cholestatic liver disease. A novel targeted mass spectrometry assay was developed for the accurate measurement of the major urinary atypical bile acids and bile alcohols that are biomarkers for HSD3B7, AKR1D1, CYP7B1 and CYP27A1 deficiencies, the four most common BASDs. **Methods:** Stable-isotope dilution UPLC tandem mass spectrometry was used for the simultaneous quantification of 12 key atypical bile acid biomarkers in urine from patients with BASD. Typical concentration ranges for these metabolites were established from urine samples from patients with biochemically and/or genetically confirmed BASD and compared with non-cholestatic and cholestatic controls. **Results:** The separation of major 3β-hydroxy-Δ^5^-bile acid sulfates, taurine- and glycine-conjugated 3-oxo-Δ^4^-bile acids, and bile alcohol glucuronides was achieved in a 20 min chromatographic run with intra- and inter-batch imprecisions of <15% for all metabolites. The mean ± SEM urinary concentration of total 3β-sulfated-Δ^5^-cholenoic acids in patients with HSD3B7 deficiency was 704 ± 204 µmol/L (n = 22), approximately 2000-fold higher than in cholestastic patients (n = 168) or non-cholestatic controls (n = 127). Similarly, the concentration of 5β-cholestane-3α,7α,12α,24,25-pentol-glucuronide, the major bile alcohol, in patients with CYP27A1 deficiency was 95 ± 17 µmol/L (n = 12). For CYP7B1 deficiency, two confirmed cases showed elevated levels (average, 7.5 µmol/L) of the glycine conjugate of 3β-sulfooxy-Δ^5^-bile acid. In AKR1D1 deficiency, total 3-oxo-Δ^4^-bile acids in urine were elevated (81 ± 16 µmol/L, n = 48), but concentrations showed overlap with cholestatic and non-cholestatic controls. **Conclusions**: A novel quantitative tandem mass spectrometry assay is described for the measurement of the major atypical metabolites and biomarkers in urine applicable to the accurate monitoring of treatment responses, and for the first time typical concentration ranges are established for each of these BASDs.

## 1. Introduction

**Figure 1:**
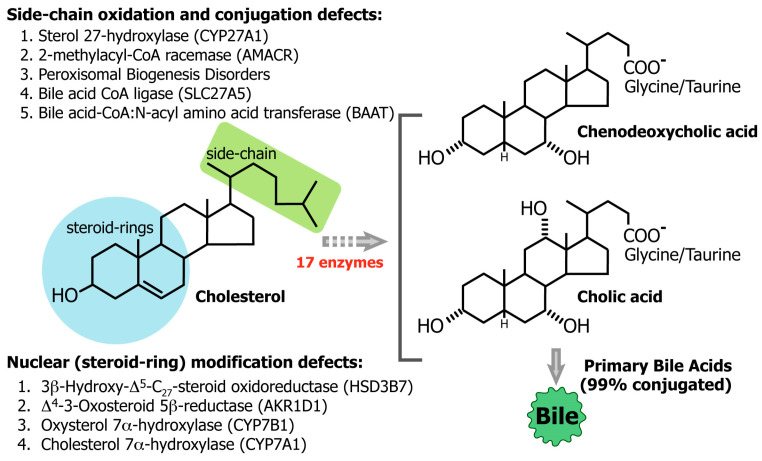
The simplified pathway for bile acid synthesis from cholesterol is depicted with the list of known genetic disorders that involve enzymes responsible for catalyzing reactions that modify the steroid ring and the side-chain of cholesterol to produce the primary bile acids of glycine- and taurine-conjugated cholic and chenodeoxycholic acids.

Bile acid synthesis disorders (BASDs), a group of autosomal recessive genetic defects, are now a well-established class of metabolic liver disease [1,2,3,4,5,6]. These disorders manifest as a broad phenotype presenting with an overlapping spectrum of variable degrees of neonatal cholestasis, fat-soluble vitamin malabsorption, and neuropathies. Diagnosis is usually made in infancy, but BASD can also account for late-onset chronic cholestasis in adolescents [7], and in some cases may be missed until adulthood. Bile acids are synthesized from cholesterol through a complex pathway involving 17 enzymes [8,9] that catalyze the conversion of this C27 neutral sterol to the C24 acidic steroids of glycine- and taurine-conjugated cholic and chenodeoxycholic acids (Figure 1). These so-called ‘primary bile acids’ provide the major driving force for the stimulation and secretion of bile, while facilitating the intestinal absorption of lipids and fat-soluble vitamins [10,11]. To date, nine defects in the cholesterol-to-bile acid biosynthetic pathway have been described [1,2,4] (Figure 1). The two most common ones, 3β-hydroxy-Δ^5^-C_27_-steroid oxidoreductase (HSD3B7) [12,13,14] and Δ^4^-3-oxosteroid 5b-reductase (AKR1D1) [14,15,16] deficiencies, and oxysterol 7α-hydroxylase (CYP7B1) deficiency [17,18,19,20] all involve enzymes that catalyze changes to the steroid rings and typically first present with idiopathic cholestasis that, undiagnosed, can be fatal. Disorders involving enzymes responsible for the modification of the cholesterol side-chain generally present with fat-soluble vitamin malabsorption or neurological or lipid disorders [21,22,23,24,25,26]. These may or may not present with cholestasis. In all cases, early diagnosis of these BASDs is crucial to prognosis because, undiagnosed and untreated, the liver disease, which is a progressive form of intrahepatic cholestasis, leads to fibrosis, cirrhosis and end-stage disease [1]. Treatment options include liver transplantation or alternatively oral bile acid therapy with the primary bile acids, cholic or chenodeoxycholic acids [27,28,29,30,31,32,33,34,35,36,37,38,39].

**Figure 2:**
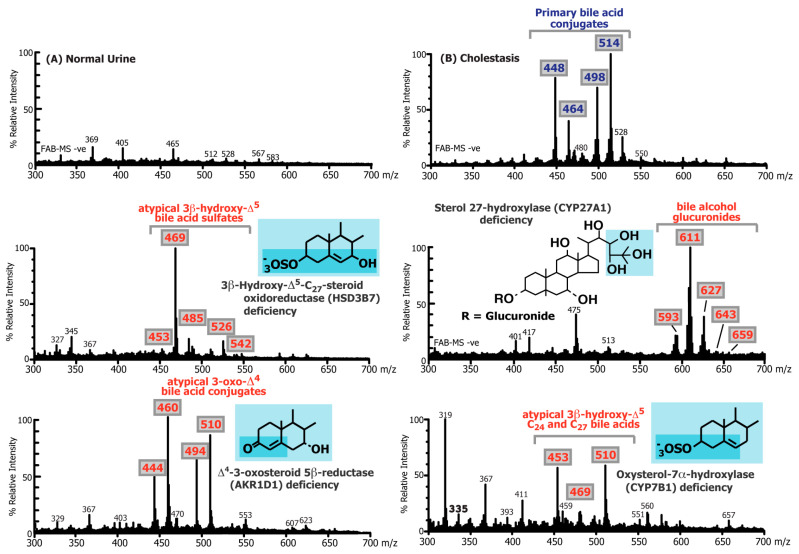
Typical urinary negative ion fast atom bombardment ionization mass spectra for patients with the four most common bile acid synthesis disorders—3β-hydroxy-Δ^5^-C_27_-steroid oxidoreductase (HSD3B7), Δ^4^-3-oxosteroid 5β-reductase (AKR1D1), oxysterol 7α-hydroxylase (CYP7B1) and sterol 27-hydroxylase (CYP27A1) deficiencies—compared with the mass spectra from healthy non-cholestatic controls and patients with cholestatic liver disease and intact bile acid synthesis.

Untargeted mass spectrometry using the secondary ionization technique of fast atom bombardment ionization mass spectrometry (FAB-MS) has been the ‘gold-standard’ approach to the diagnosis of BASD for 40 years [1,40,41]. Diagnosis is based upon the detection of elevated levels of specific atypical bile acids or intermediates that accumulate in urine resulting from of a lack of enzyme activity caused by mutations in the genes encoding these enzymes [42,43,44,45,46] (Figure 2). Even though genetic tests are now available, the long turnaround time for results may compromise the clinical course or outcome. Genetic testing can however be helpful in identifying mutations in the genes and in our experience is best used as a confirmatory test for the biochemical identification of these BASD through mass spectrometry. While FAB-MS continues to be used in our screening program for the diagnosis and monitoring of response to therapy, it is an obsolescent technique, only semi-quantitative, and unable to accurately quantify concentrations of bile acids in urine. Electrospray ionization (ESI) tandem mass spectrometry offers an alternative approach to quantify these atypical bile acid species [41,47,48,49,50,51,52,53,54,55,56].

Accurate quantification of atypical bile acids in biological fluids has until recently proven problematic because of the lack of available authentic reference standards and stable-isotope-labeled internal standards that are necessary to develop a robust quantitative assay. We now describe a novel targeted assay using stable-isotope dilution tandem mass spectrometry for the measurement of the major atypical bile acids and bile alcohols that are the specific biomarkers for HSD3B7, AKR1D1, CYP7B1 and CYP27A1 deficiencies. Furthermore, following the FDA approval of Cholbam (cholic acid) for the treatment of BASD, and more recently chenodeoxycholic acid for CTX, this assay will permit a more accurate evaluation of the biochemical response to bile acid therapy while allowing for drug dosing adjustments to be made based on the extent of suppression of atypical bile acid concentrations.

## 2. Materials and Methods

### 2.1. Chemicals, Reagents and Reference Compounds

Reference compounds of the major atypical bile acids and sterols, which are the diagnostically important biomarkers for each of the bile acid synthesis disorders, and the corresponding stable-isotope-labeled internal standards were custom synthesized by IRBM Science S.p.A. (Pomezia, Italy). The following reference compounds were obtained for each of the disorders: (a) 3β-hydroxy-Δ^5^-C_27_-steroid oxidoreductase (HSD3B7) deficiency: the sulfate conjugates of 3β,7α-dihydroxy-5-cholen-24-oic acid (HSD-469) and 3β,7α,12α-trihydroxy-5-cholen-24-oic acid (HSD-485) and the glyco-sulfate conjugates of 3β,7α-dihydroxy-5-cholen-24-oic acid (HSD-526) and 3β,7α,12α-trihydroxy-5-cholen-24-oic acid (HSD-542); (b) Δ^4^-oxosteroid 5β-reductase (AKR1D1) deficiency: the taurine conjugates of 3-oxo-7α-hydroxy-chol-4-en-24-oic acid (AKR-494) and 3-oxo-7α,12α-dihydroxy-chol-4-en-24-oic acid (AKR-510) and the glycine conjugates of 3-oxo-7α-hydroxy-chol-4-en-24-oic acid (AKR-444) and 3-oxo-7α,12α-dihydroxy-chol-4-en-24-oic acid (AKR-460); (c) oxysterol 7a-hydroxylase (CYP7B1) deficiency: the sulfate conjugate of 3β-hydroxy-5-cholen-24-oic acid (CYP7-453) and the glycine conjugate of 3β-hydroxy-5-cholen-24-oic acid (CYP7-510); (d) sterol 27-hydroxylase (CYP27A1) deficiency: 5β-cholestane-3α,7α,12α, 25-tetrol-3-O-β-glucuronide (CTX-611) and 5β-cholestane-3α,7α,12α, 23S, 25-pentol-23-O-β-glucuronide (CTX-627). All solvents, reagents and chemicals were of analytical-grade purity and were obtained from Sigma-Aldrich (St. Louis, MO, USA) and Thermo Fisher Scientific (Waltham, MA, USA). Urine used for the biological matrix in the preparation of calibrators and quality control samples was obtained from UTAK (Valencia, CA, USA).

### 2.2. Urine Samples from Patients with Bile Acid Synthesis Disorders

Urine samples, with volumes ranging from 0.1 to 25 mL, were obtained from patients with idiopathic cholestasis (n = 168 (167 with age data), mean age 13.5 months, range 0.1–608.2 months, median 1.4 months), from non-cholestatic controls (n = 127, mean age 204.1 months, range 0–717.3 months, median 150.3) and from patients that had a confirmed diagnosis of HSD3B7 (n = 22 (21 with age data), mean age 74.4 months, range 3.5–386.9 months, median 37.1), AKR1D1 (n = 48 (47 with age data), mean age 10.1 months, range 0.1–194.2 months median 4.1), CYP7B1 (n = 2, mean age 92.4 months, 5.5 and 179.4 months) and CYP27A1 (n = 21, mean age 172.2 months, range 2.9–528.7 months, median 115.6 months) deficiency. Age data were missing for total three patients due to incomplete clinical records. Biomarker measurements were available for all patients and were used for group comparisons. This study was performed on de-identified, to-be-discarded urine obtained after clinical investigations were completed. As only fully anonymized patient samples were used that were not obtained specifically for use in this study through an interaction or intervention with living individuals, neither informed consent nor Institutional Review Board (IRB) review were required. The only information provided with the deidentified samples was the confirmed diagnosis, which had been established biochemically by urine FAB-MS analysis and/or genetic confirmation, and the patient’s age in order to obtain representative clinical ranges for the key atypical metabolites in these bile acid synthesis disorders.

### 2.3. Preparation of Reference Standards and Samples for LC-MS/MS Analysis

Calibration standards were prepared from the stock solutions (1 mg/mL in methanol/water, 1:1, *v*/*v*) of the atypical reference compounds by serial dilution to achieve final concentrations of 0, 25, 50, 200, 500, 1000, 2500, and 5000 ng/mL. Quality control (QC) samples were prepared by spiking the reference compounds to a UTAK pooled urine matrix to achieve concentrations of 100, 400, and 2000 ng/mL, representing QC-Low, QC-Med, and QC-High concentrations, respectively. All calibrators and QC samples were stored at −20 °C. A patient QC urine pool was prepared by mixing 15 urine samples from de-identified patient samples with known bile acid synthesis defects (BASDs), and this was included in each analytical run.

The stable-isotope-labeled internal standards comprised a mixture of 11 compounds dissolved in methanol (1 mg/mL). A 100 µL volume was added to 100 µL of all calibrators, QC samples and urine from healthy subjects and patients with established bile acid synthesis disorders. The samples were vortexed for 10 secs followed by the addition of MilliQ H_2_O (1.0 mL), and bile acids were extracted through solid phase extraction (SPE) on a 3 mL C18-E cartridge that was first pre-charged by sequential washes with HPLC-grade methanol (2 mL) and MilliQ water (2 mL) [57,58]. The sample was transferred to the C18-E cartridge and slowly pulled through under vacuum at a flow rate of approximately 1 drop per second, and the eluent was discarded. The cartridge was rinsed with 2 mL of MilliQ water, and bile acids were recovered through elution with methanol (2.0 mL) under gravity. The methanol eluant was dried under a stream of nitrogen gas at 60 °C then reconstituted in 200 µL of 50% methanol/H_2_O, and 10 µL was injected onto a column for analysis.

### 2.4. FAB-MS Analysis of Urine

The diagnosis of a BASD in patients was established from clinical features and following a routine-validated FAB-MS of the urine that has been in clinical practice for >35 years. In FAB-MS negative ion mode, prominent deprotonated molecular ions are observed in the mass spectrum (Figure 2) that reflect the presence of elevated levels of the atypical biomarkers that definitively confirm the biochemical abnormality [2]. These ions provided the basis for establishing the quantitative LC-ESI-MS/MS assay described here.

### 2.5. Analysis of Atypical Bile Acid and Sterol Biomarkers Using LC-ESI-MS/MS

The quantitative analysis of 12 atypical metabolites that definitively characterize these BASDs was performed through ultra-high-performance liquid chromatography electrospray ionization–tandem mass spectrometry (LC-ESI-MS/MS) using a Waters TQ-XS triple quadruple mass spectrometer interfaced with an Equity HPLC system (Milford, MA). The quantification of atypical bile acids in human urine was achieved using LC-MS analysis with multiple-reaction monitoring (MRM) under negative ion mode detection. The mass spectrometry instrument settings and conditions, including ion source temperature, desolvation gas flow, cone voltages and ionization energy, etc., are detailed in the Supplementary Materials (Table S1). Chromatographic separation of the individual atypical metabolites was achieved on a reverse-phase octadecylsilane (C18) column (Thermo Hypersil™ BDS C18, 3 µm, 100 × 2.0 mm; Thermo, Waltham, MA, USA). Mobile phase A was 20% Acetonitrile/water with 10 mM Ammonium Acetate and mobile phase B was 80% Acetonitrile/water with 10 mM Ammonium Acetate. A multi-step gradient was used starting at 5% B: from 0.4 to 5 min, it was ramped to 15% B; then, from 5 to 10 min, it increased to 25% B; from 10 to 12 min, it ramped to 50% B; then, from 12 to 14 min, it increased to 75% B; and from 14 to 16 min it increased to 100% B. After maintaining 100% B for 4 min, it was returned to the initial gradient condition. The mobile-phase flow rate was 0.4 mL/min.

### 2.6. Statistical Analysis

Atypical bile acids were compared across clinical samples using one-way analysis of variance (ANOVA). Due to the unbalanced design, Type II sums of squares were employed to account for unequal group sizes without sequential order effects [59]. Prior to analysis, assumptions were assessed: the normality of residuals was evaluated using the Shapiro–Wilk test, and homogeneity of variances was tested using Levene’s test. Effect sizes were quantified using omega-squared (ω^2^) to provide an unbiased estimate of population effect size given the unequal sample sizes. Omega-squared values of 0.01, 0.06, and 0.14 were interpreted as small, medium, and large effects, respectively [60]. When the overall ANOVA was significant (*p* < 0.05), post hoc pairwise comparisons were conducted. If variances were homogeneous, Tukey’s Honest Significant Difference (HSD) test was used to control the family-wise error rate. If variances were heterogeneous (Levene’s test *p* < 0.05), the Games–Howell test, which does not assume equal variances or sample sizes, was employed. Sensitivity analyses included Welch’s ANOVA (robust to unequal variances). All analyses were performed using R version 4.5.2. A two-tailed *p*-value < 0.05 was considered statistically significant. Receiver operating characteristic (ROC) curve analysis was performed to evaluate the diagnostic performance of urinary atypical bile acid metabolites for distinguishing patients with bile acid synthetic defects from controls. ROC curves were generated using biomarker concentrations as continuous variables, and the area under the curve (AUC) was calculated to assess overall discriminative ability [61]. Optimal cutoff values were estimated using Youden’s index to balance sensitivity and specificity. Given the rarity of bile acid synthetic defects and the limited sample size, the ROC analysis was intended as an exploratory assessment of diagnostic utility rather than definitive clinical validation. Confidence intervals for AUC, sensitivity, and specificity were calculated where appropriate. These analyses support the potential utility of urinary atypical bile acid metabolites as candidate diagnostic biomarkers and provide a framework for future validation in independent cohorts.

## 3. Results and Discussion

### 3.1. Optimization of a Quantitative LC-ESI-MS/MS Method

**Figure 3:**
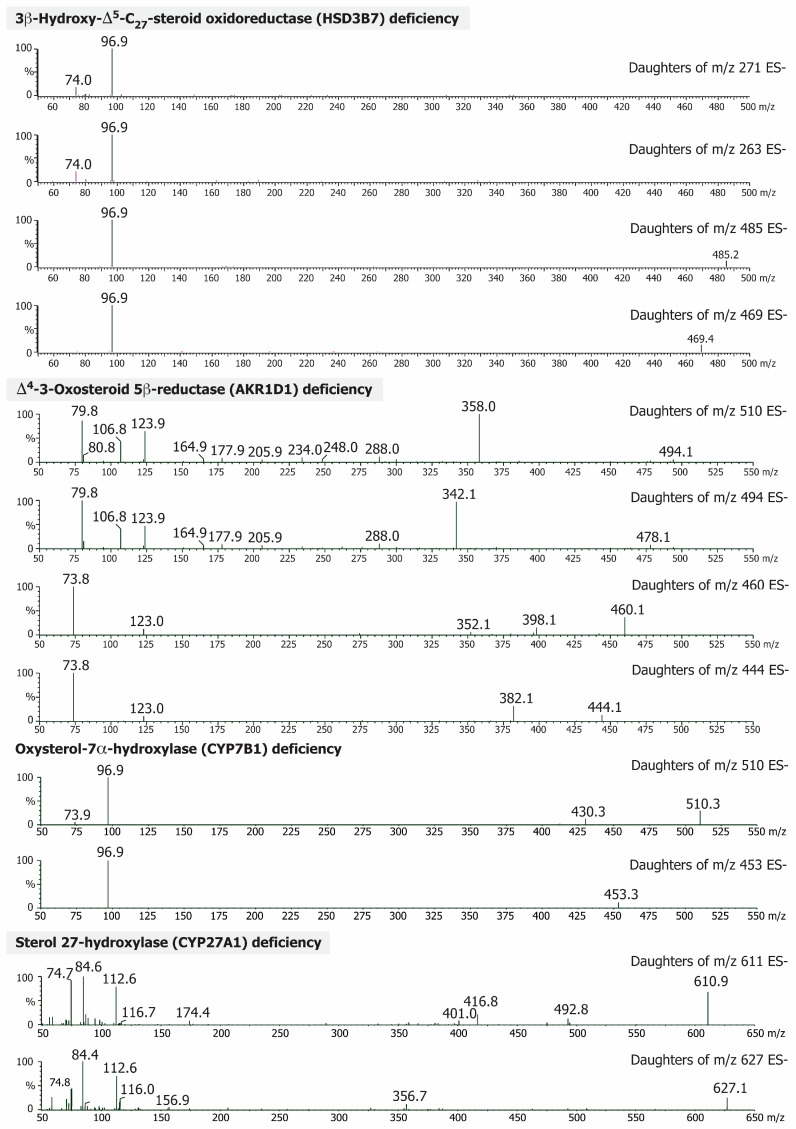
The negative ion MS/MS collision-induced dissociation mass spectra for the reference compounds of the major atypical metabolites monitored for the HSD3B7, AKR1D1, CYP7B1 and CYP27A1 deficiencies.

**Figure 4:**
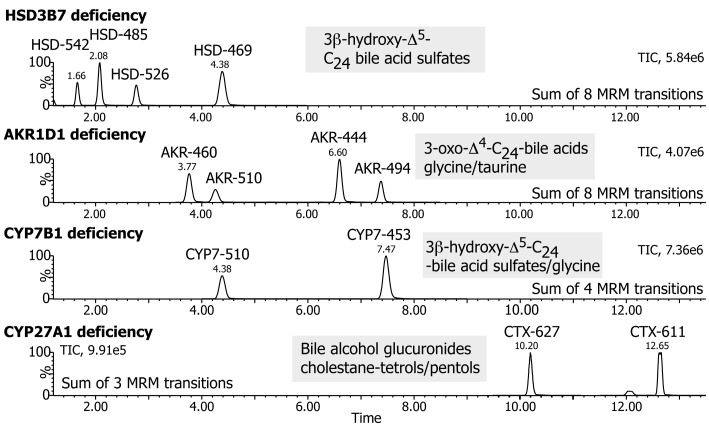
HPLC-MS chromatographic separation of the key bile acid and sterol intermediates, the biomarkers for the bile acid synthesis disorders of 3β-hydroxy-Δ^5^-C_27_-steroid oxidoreductase (HSD3B7 deficiency, Δ^4^-3-oxosteroid 5β-reductase (AKR1D1) deficiency, oxysterol 7α-hydroxylase (CYP7B1) deficiency, sterol 27-hydroxylase (CYP27A1) deficiency, and cerebrotendinous xanthomatosis (CTX). Shown are the total ion current for the summed MRM transitions and the respective internal standards used to quantify each biomarker.

The assay permits the simultaneous quantification of the diagnostically significant biomarkers for HSD3B7, AKR1D1, CYP7B1 and CYP27A1 deficiencies [1] in a single analysis. The choice of negative ions for monitoring was based on features of the FAB-MS negative ion mass spectrum (Figure 2) and the designated ions that define the individual BASD. The negative ion collision-induced dissociation (CID) mass spectrum for the deprotonated molecular ion of each reference compound is shown in Figure 3, and the optimized MRM transitions selected to monitor each atypical metabolite and the respective stable-isotope-labeled internal standard are listed in Table 1. Chromatographic separation of all 12 atypical bile acid and sterol biomarkers was achieved through reverse-phase chromatography with baseline resolution using a Thermo C18 column and gradient elution with a total run time of 20 min per sample (Figure 4).

#### 3.1.1. 3β-Hydroxy-Δ^5^-C_27_-Steroid Oxidoreductase (HSD3B7) Deficiency

The negative ion FAB-MS spectrum of the urine from patients with HSD3B7 deficiencies [12] features prominent ions at *m*/*z* 469 and 485, which represent the deprotonated molecular ions [M-H]^−^ of the sulfate conjugates of 3β,7α-dihydroxy-5-cholen-24-oic and 3β,7α,12α-trihydroxy-5-cholen-24-oic acids, respectively, and the corresponding glyco-sulfate-conjugated species at *m*/*z* 526 and 542 [41]. In some patients, sulfated monohydroxylated 3β-hydroxy-5-cholen-24-oic bile acid (*m*/*z* 453) may be present at low levels. The presence of these ions is definitive for a diagnosis of HSD3B7 deficiency. These atypical bile acids were selected for monitoring with ESI-MS/MS. The electrospray ionization negative ion mass spectra of these atypical bile acids have been reported previously [12,41]. The sulfate conjugates 3β,7α-dihydroxy-5-cholen-24-oic acid (designated HSD-469) and 3β,7α,12α-trihydroxy-5-cholen-24-oic acid (HSD-485) both reveal intense singly charged deprotonated ions [M-H]^−^ with little fragmentation. Likewise, the corresponding glyco-sulfate conjugates (designated as HSD-526 and HSD-542) also yield singly charged deprotonated ions at *m*/*z* 526.2 and 542.2. However, due to the double conjugate structure (side-chain and steroid ring), these atypical bile acids also generated doubly charged ions at *m*/*z* 262.7 and 270.7, respectively. The cone energy was therefore optimized at 30 eV to generate mainly deprotonated ions. Under collision-induced dissociation (CID) the parent ions yield a common intense base peak at *m*/*z* 96.9 resulting from loss of the sulfate group ([HSO_4_^−^]). Consequently, the deprotonated molecular ions and the corresponding CID product ions were selected as the optimal mass transition ion pairs for monitoring and quantification of all the 3β-hydroxy-Δ^5^-bile acid sulfates (Figure 3). The corresponding stable-isotope-labeled internal standards gave similar fragmentation patterns, and thus the MRM transitions optimized for the MS parameters and monitored were at *m*/*z* 474.3 > 97.9, 490.2 > 97.8, 265.5 > 96.9, 273.3 > 96.9 for HSD-469-IS, HSD-485-IS, HSD-526-IS, and HSD-542-IS, respectively (Table 1). The internals standards HSD-469-IS and HSD-485-IS had a predominant fragment ion of *m*/*z* 97.9, which corresponds to [HSO_4_^−^] plus a deuterium transfer, most likely from the deuterium atom at the C-3 position. On the other hand, HSD-526-IS and HSD-542-IS had a predominant fragment of only *m*/*z* 96.9.

#### 3.1.2. Δ^4^-3-Oxosteroid 5β-Reductase (AKR1D1) Deficiency

With a reduced activity of AKR1D1, hepatotoxic and cholestatic 3-oxo-Δ^4^- bile acids accumulate and are excreted in urine [15]. The negative ion FAB-MS spectrum of the urine from patients with AKR1D1 deficiency (Figure 2) is characterized by ions at *m*/*z* 444 and 460 for the glycine conjugates and *m*/*z* 494 and 510 for the taurine conjugates of 3-oxo-7α-hydroxy-chol-4-en-24-oic and 3-oxo-7α,12α-dihydroxy-chol-4-en-24-oic acids, respectively [1,15]. These are the biomarkers for AKR1D1 deficiency and were selected for monitoring with ESI-MS/MS. Under ESI, these atypical 3-oxo-Δ^4^- bile acids (designated AKR-444, AKR-460, AKR-494 and AKR-510) yielded intense, predominantly singly charged deprotonated molecular ions [M-H]^−^ at *m*/*z* 444.2, 460.2, 494.2 and 510.2, respectively. On collision-induced dissociation of the parent ions, the glycine conjugates of AKR-444 and AKR-460 fragmented to yield an intense base peak at *m*/*z* 73.8 due to the loss of glycine, while the analogous fragmentation of the taurine conjugates of AKR-494 and AKR-510 gave an intense base peak at *m*/*z* 79.8. The taurine conjugates required higher collision energy (Table 1) to generate optimal fragmentation when compared with the glycine conjugates. AKR-494 and AKR-510 yielded predominant ions at *m*/*z* 342.1 and *m*/*z* 358.1, arising from cleavage across the AB/CD rings of the steroid nucleus, and these were selected for monitoring and quantification. The internal standards for the glycine conjugates (AKR-444-IS, AKR-460-IS) were labeled with a combination of two [^13^C] atoms at the positions of the C-1 and C-2 carbons of the glycine moiety, a [^15^N] atom on the amino group, and two [^2^H] atoms at position C-2 of the glycine moiety, giving a total mass shift of +5 Da for the parent ion mass. Consequently, the fragmentation of these two glycine-conjugated ISs generated a fragment of *m*/*z* 78.9 for loss of the labeled glycine, reflecting the 5Da mass shift. The taurine-conjugated internal standards (AKR-494-IS, AKR-510-IS) were labeled with [^2^H] atoms at positions C-1 and C-2 of the taurine moiety, thus increasing the deprotonated molecular ion by 4Da. On CID fragmentation, *m*/*z* 79.8 was the predominant ion formed, together with *m*/*z* 346.1 and *m*/*z* 362.1, from cleavage across the steroid rings (Figure 3). For greater selectivity and optimal sensitivity, *m*/*z* 498.2 > 346.1 and 514.2 > 362.0, respectively, were selected as the transition ion pairs for monitoring the internal standards AKR-494-IS and AKR-510-IS.

#### 3.1.3. Oxysterol 7α-Hydroxylase (CYP7B1) Deficiency

This bile acid synthesis disorder is caused by mutations in the *CYP7B1* gene and presents as a severe and usually fatal cholestatic disease because of the accumulation of the hepatotoxic monohydroxy bile acid 3β-hydroxy-chol-5-enoic acid [17]. Interestingly, it has also been associated with hereditary spastic paraplegia, a degenerative motor neuron condition [62,63]. Biochemically, it presents with elevated levels of sulfate and glyco-sulfate conjugates of the monohydroxylated 3β-hydroxy-chol-5-enoic acid in urine (Figure 2). The negative ion ESI mass spectra of these biomarkers (designated CYP7-453 and CYP7-510, respectively) show intense singly charged deprotonated ions [M-H]^−^ at *m*/*z* 453.3 and 510.3, respectively. With collision-induced dissociation of the parent ions, both metabolites yielded a common intense base peak at *m*/*z* 96.9 (Figure 3) from loss of the sulfate group, and this MRM transition was used. The internal standards were labeled in the steroid nucleus and side-chain of the sulfate conjugate ([2,3,3,23,23-^2^H_5_]3β-sulfooxy-chol-5-en-24-oic acid), and in the glycine moiety ([1,2-^13^C2, 2,2-^2^H_2_, 3-^15^N]glycine) for the corresponding glyco-sulfate conjugate. ESI mass spectra yielded analogous spectra to the pure reference unlabeled standards, with mass shifts based on the positions of the stable-isotopic atoms (Table 1). These standards were used to optimize the MS parameters, and the MRM transitions selected for monitoring were *m*/*z* 458.3 > 97.9, 515.4 > 96.9, respectively. The internal standard CYP7-453-IS had a predominant fragment at *m*/*z* 97.9, analogous to that observed for HSD-469-IS and HSD-485-IS.

#### 3.1.4. Sterol 27-Hydroxylase (CYP27A1) Deficiency

This BASD presents as the rare lipid storage disease of cerebrotendinous xanthomatosis (CTX) [43,64,65]. Patients with CYP27A1 deficiency excrete very high concentrations of polyhydroxylated bile alcohol glucuronides in urine [66,67] that are readily detected through FAB-MS from the intense deprotonated molecular ion [40,42] (Figure 2). The major urinary bile alcohol glucuronides are 5β-cholestane-3α,7α,12α, 25-tetrol-3-O-β-glucuronide (designated CTX-611) and 5β-cholestane-3α,7α,12α, 23S, 25-pentol-23-O-β-glucuronide (CTX-627) [68]. ESI of the reference compounds yielded intense deprotonated ions [M-H]^−^ at *m*/*z* 611.0 and 626.9, respectively. With collision-induced dissociation of the parent ion, both yielded a common intense base peak at *m*/*z* 84.7, together with two other fragments at *m*/*z* 74.7 and *m*/*z* 112.7 (Figure 3). For quantification of these bile alcohol glucuronides, the only reference internal standard available was the [26,26,26,27,27,27-^2^H_6_]5β-cholestane-3α,7α,12α, 25-tetrol-3-O-β-glucuronide (CTX-611-IS). On CID, fragment ions at *m*/*z* 84.7, *m*/*z* 74.7 and *m*/*z* 112.7 were generated as was observed for the unlabeled reference standard.

### 3.2. Assay Performance and Validation

A full validation of the assay, including linearity, within- and between-batch imprecision and accuracy, freeze–thaw cycle, dilution integrity, and short- and long-term stability, was performed, and the assay was found to be highly specific, reproducible and robust. Calibration curves for all the atypical bile acids and sterols were linear over the dynamic range of 25 to 5000 ng/mL. The lower limit of quantification (LLOQ), taken as the lowest concentration measurable in urine with a coefficient of variation (CV) of <20%, was 50 ng/mL for all metabolites (Figures S1–S4). The recovery for 12 analytes from pooled urine was high but varied among the metabolites, with the bile alcohol glucuronides showing a lower recovery than other bile acids. The recoveries of the metabolites HSD-469, HSD-485, HSD-526, HSD-542, AKR-444, AKR-460, AKR-494, AKR-510, CYP7-453, CYP7-510, CTX-611 and CTX-627 ranged from 65 to 98% (Table S2).

Three QC samples of different concentrations of atypical metabolites (QC-Low 100, QC-Med 400, and QC-High 2000 ng/mL) spiked to a urine matrix were included within each batch of samples assayed. The recoveries of the atypical metabolites from these quality control samples ranged from 65.4 and 99.9%, and the within-run % CV and % bias for all the atypical bile acids was <13% (Table 2). The between-batch imprecision based on five separate batches of assays, expressed as % CV and % bias for all the atypical bile acids, was <10% (Table 2). The matrix effect was evaluated by comparing the response of post-spiked standards in a blank matrix extract to that of pre-spiked samples. The matrix effect factor was in the range of 0.7–1.0 for all atypical bile acids (Table S3).

The stability of the atypical bile acids was determined from the analysis of urine spiked with the reference standards at concentrations of 100 and 2000 ng/mL, respectively, for the QC-Low and QC-High samples. After three freeze–thaw cycles of the QC-Low and QC-High samples, the % differences ranged from −1.9 to 7.9% and −1.9 to 8.2%, respectively, at −20 °C. All the atypical bile acids were stable for 6h at ambient temperature, after 24h in the autosampler, and when stored for three months at −20 °C.

### 3.3. Clinical Application of LC-ESI-MS Method

**Figure 5:**
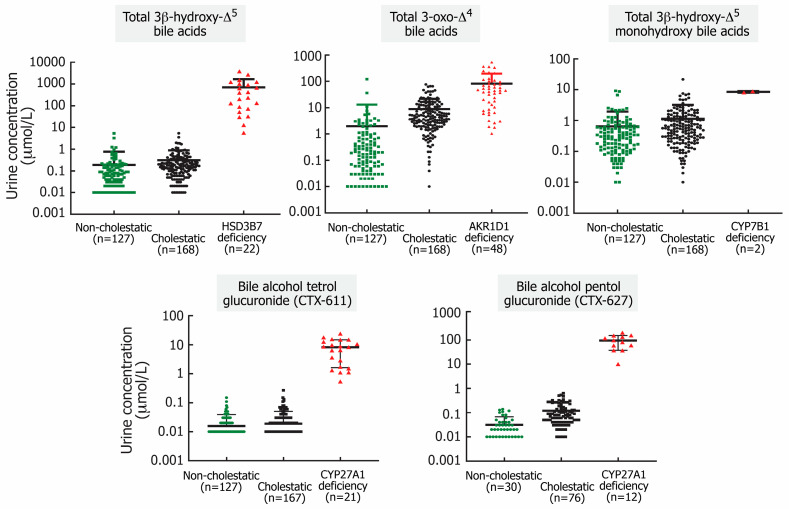
Urinary bile acid concentrations of the major atypical bile acids and sterols that represent the biomarkers of HSD3B7, AKR1D1, CYP7B1 and CYP27A1 deficiencies.

This analytical method was applied to determine the concentrations of atypical bile acids and sterols in randomly collected urines from a total of 93 patients with documented BASD, 168 patients with idiopathic liver disease that were found to be negative for a BASD, and 127 non-cholestatic controls. The patients with BASD comprised 22 patients with HSD3B7 deficiency, 48 patients with AKR1D1 deficiency, two patients with CYP7B1 deficiency and 21 patients with CYP27A1 deficiency. From these data, ranges for the atypical metabolites were established, and clinical ranges were determined for those patients with confirmed BASD (Table 3; Figure 5).

#### 3.3.1. 3β-Hydroxy-Δ^5^-C_27_-Steroid Oxidoreductase (HSD3B7) Deficiency

We previously reported the novel chemical synthesis of the sulfate and glyco-sulfate forms of 3β,7α-dihydroxy-chol-5-en-24-oic and 3β,7α,12α-trihydroxy-chol-5-en-24-oic acids [41] and described a tandem mass spectrometric method for the direct quantification of these sulfate conjugates in urine. This previously described assay used UDCA-7-sulfate as the internal standard because, at the time, stable-isotopically labeled standards were unavailable. Now, with the availability and inclusion of the four stable-isotope-labeled internal standards, the assay has been modified and improved. The mean ± SEM urinary concentration of the total sulfated 3β-hydroxy-Δ^5^-cholenoic acids (the sum of all four metabolites) in patients with a confirmed HSD3B7 deficiency was 704 ± 204 µmol/L (n = 22), approximately 2000-fold higher than in cholestastic patients with intact primary bile acid synthesis (0.31 ± 0.04 µmol/L, n = 168) or non-cholestatic controls (0.19 ± 0.05 µmol/L, n = 127) (Figure 5). HSD3B7 patients and controls differed significantly in sample size (non-cholestatic controls: n = 127, cholestatic control: n = 168, HSD3B7 patients: n = 22). Levene’s test indicated that the assumption of homogeneity of variances was not met (*p* < 0.001). One-way ANOVA with Type II sums of squares revealed a statistically significant difference in total 3β-hydroxy-Δ^5^-bile acids across the three cohorts (F(2, 314) = 82.57, *p* < 0.001, ω^2^ = 0.34), indicating a large effect size according to Cohen’s guidelines. Post hoc comparisons using the Games–Howell test showed that HSD3B7 patients had significantly higher 3β-hydroxy-Δ^5^-bile acids compared to both controls (*p* = 0.007). No significant differences were observed between two control groups (*p* = 0.185). These findings were consistent in sensitivity analyses using Welch’s ANOVA (F(2, 52.0) = 7.4, *p* = 0.002). The variability in the concentration of the individual 3β-hydroxy-Δ^5^-bile acids can be explained by the extent of liver dysfunction and age of the patient at the time of diagnosis (Table 3). ROC curve analysis showed that urinary atypical bile acid metabolites effectively discriminated patients with HSD3B7 deficiency from both cholestatic and non-cholestatic control groups. Both HSD-469 and total HSD demonstrated excellent diagnostic performance, with AUC values of 1.0 in comparison with controls. Using Youden’s index, preliminary cutoff values were identified at 0.765 µmol/L and 5.46 µmol/L for HSD-469 and total HSD, respectively, when compared with cholestatic controls, and at 0.925 µmol/L and 5.44 µmol/L when compared with non-cholestatic controls (Figures S5 and S6).

#### 3.3.2. Δ^4^-Oxosteroid 5β-Reductase (AKR1D1) Deficiency

Diagnosis of a AKR1D1 deficiency [15] remains the most challenging of all the BASDs because the atypical 3-oxo-Δ^4^- bile acids that are the diagnostic biomarkers are normally excreted in urine in early life [69,70,71]. This is a consequence of an immaturity in hepatic bile acid synthesis and bile acid transport, reflected by a natural physiologic cholestasis observed in all neonates [72,73]. AKR1D1 enzyme activity is also impacted in advanced liver disease when there is significant loss of synthetic function, so that 3-oxo-Δ^4^- bile acids may be found in increased concentrations in end-stage disease or other metabolic disease [74,75,76]. Delineating whether an increase in 3-oxo-Δ^4^- bile acids in urine reflects a ‘primary’ genetic defect or is ‘secondary’ to these scenarios [77] is facilitated by complementing the biochemical test with genetic testing for variants in *AKR1D1* [78,79,80]. Irrespective of this, the presence of elevated levels of 3-oxo-Δ^4^- bile acids reflects a deficiency in the activity of the AKR1D1 enzyme. Diagnosis has been achieved using FAB-MS analysis of urine from the presence of dominant ions at *m*/*z* 444, 460, 494 and 510 in the negative ion mass spectrum (Figure 2). [1,15,81] These ions and the MRM transitions generated on CID were consequently selected for quantification in this tandem MS assay. Urine from patients with genetically confirmed AKR1D1 deficiency prior to any bile acid therapy had relatively high concentrations of 3-oxo-Δ^4^- bile acids. The mean ± SEM urine concentrations for the total 3-oxo-Δ^4^- bile acids were 81.4 ± 16.3 µmol/L. For the glycine conjugate of 3-oxo-7α-hydroxy-chol-4-en-24-oic acid (AKR-444), the mean concentration was 9.4 ± 1.4 µmol/L (n = 48), which was 12- and 24-fold greater than that of patients with cholestasis (0.8 ± 0.2 µmol/L, n = 168) and non-cholestatic controls (0.4 ± 0.4 µmol/L, n = 127) (Table 3). However, there was some overlap among the groups (Figure 5), making definitive diagnosis of an AKR1D1 deficiency exclusively based on urine 3-oxo-Δ^4^- bile acid concentration difficult and further supporting the need for complementary genetic testing in the differential diagnosis of an AKR1D1 deficiency. One-way ANOVA with Type II sums of squares showed a statistically significant difference in total 3-oxo-Δ^4^- bile acids (*p* < 0.001). Post hoc comparisons using the Games–Howell test showed that patients with AKR1D1 deficiency had significantly higher 3-oxo-Δ^4^- bile acids compared to both controls (*p* < 0.001). Significant differences were also observed between two control groups (*p* < 0.001). These findings were consistent in sensitivity analyses using Welch’s ANOVA (F(2, 52.0) = 7.4, *p* = 0.002). ROC curve analysis demonstrated that urinary atypical bile acid metabolites effectively discriminated patients with AKR1D1 deficiency from non-cholestatic controls, with excellent diagnostic performance for both biomarkers (AUC = 0.984 [95% CI: 0.963–0.998] for AKR-444 and 0.975 [95% CI: 0.953–0.990] for total AKR). Using Youden’s index, preliminary cutoff values of 0.37 µmol/L for AKR-444 and 4.64 µmol/L for total AKR achieved a balance between sensitivity and specificity. When compared with cholestatic controls, diagnostic performance was achieved, with AUC values of 0.917 (95% CI: 0.865–0.954) for AKR-444 and 0.839 (95% CI: 0.760–0.904) for total AKR, corresponding to preliminary cutoff values of 1.06 µmol/L and 31.5 µmol/L, respectively (Figures S5 and S6). Although confidence intervals reflected some uncertainty due to limited sample size, the discriminatory trends were consistent across all atypical bile acid metabolites examined. Taken together, these findings support the potential diagnostic utility of urinary atypical bile acid metabolites and highlight their promise as candidate biomarkers, while underscoring the need for validation in larger, independent cohorts.

In addition, the ROC analysis provides a valuable benchmark for monitoring patient response to treatments, even with its limitations. For instance, for the AKR1D1 deficiency, the cutoff against non-cholestatic controls (0.37 µmol/L for AKR-444 and 4.64 µmol/L for total AKR) could serve as a target for biochemical remission or detecting relapse. Once a treated patient’s levels fall below this threshold, their biomarker profile is indistinguishable from healthy individuals. If it later shows levels rising above that threshold, it could indicate non-compliance to therapy and trigger investigation for treatment failure or disease recurrence. Therefore, this is a clinically meaningful endpoint. More importantly, these cutoffs provide a preliminary reference range for other centers during therapeutic monitoring.

#### 3.3.3. Oxysterol 7a-Hydroxylase (CYP7B1) Deficiency

Patients with CYP7B1 deficiency lack the normal primary bile acid conjugates and excrete increased concentrations of monohydroxy 3β-hydroxy-Δ^5^ bile acids [17] (Figure 1). This enzyme is critical in the alternative ‘acidic’ pathway for bile acid synthesis and is required to detoxify the highly hepatotoxic 3β-hydroxy-Δ^5^ bile acids that accumulate. Diagnosis of a CYP7B1 deficiency has been based on urine analysis and the finding of prominent ions at the *m*/*z* of 453 and 510 for the sulfate and glyco-sulfate conjugates (Figure 2). The availability of two CYP7B1 deficiency biomarkers, 3β-hydroxy-5-cholenoic acid sulfate (CYP7-453) and the glyco-sulfate conjugate (CYP7-510), plus the corresponding isotope-labeled standards, permitted the development of a quantitative assay for CYP7B1 deficiency.

Among the bile acid synthesis defects we have screened for over a 40+ year period, CYP7B1 deficiency is the least common, and this may be because it presents early and with the most severe cholestasis [19,34]. Other than the index case reported previously [17], we have documented only two cases, both from the same family in China. Quantification of the urinary concentration of 3β-hydroxy-5-cholenoic glyco-sulfate (CYP7-510) confirmed marked elevations in concentration at baseline: 6.10 µmol/L for the proband and 8.8 µmol/L for her brother (mean ± SEM). The normal range for non-cholestatic patients was 0.63 ± 0.13 µmol/L (n = 127) and for cholestatic patients was 1.10 ± 0.21 µmol/L (n = 168) (Table 3). ANOVA analysis was not conducted because the interpretation of these findings is limited by the very small sample size.

#### 3.3.4. Sterol 27-Hydroxylase (CYP27A1) Deficiency

Variants in *CYP27A1* manifest as the rare lipid storage disease of cerebrotendinous xanthomatosis (CTX) [43,65,82,83,84]. This has a broad clinical presentation and is typically not diagnosed until the second or third decades of life (average age at diagnosis is 34 years), by which time significant accumulation of cholesterol and cholestanol has occurred, leading to the symptomology [85,86,87]. Early diagnosis is critical for implementing primary bile acid therapy to slow the progression of the disease [88]. Liver disease is generally not listed as a feature of the disease, although a transient cholestasis may be observed in the first few months of life [89,90,91,92]. In some patients, cholestasis can be fatal or lead to liver transplantation [42,93]. Sterol 27-hydroxylation is a critical step in the shortening of the C8 sterol side-chain to enable oxidation in the peroxisome to produce primary bile acids. The diagnosis of CTX is readily established from markedly elevated levels of bile alcohol glucuronides [40,42,53,91,94,95] and cholestanol [96], or the sterol intermediate 7α-hydroxy-4-cholesten-3-one (C4) [55,56,97]. Tetrahydroxy-, pentahydroxy- and hexahydroxy-bile alcohol glucuronides (*m*/*z* 611, 627, and 643, respectively) are the dominant diagnostic biomarkers in the negative ion FAB-MS spectrum of the urine of patients with CTX. In this tandem MS assay, 5β-cholestane-3α,7α,12α,25-tetrol-glucuronide (*m*/*z* 611) and 5β-cholestane-3α,7α,12α,23,25-pentol-glucuronide (*m*/*z* 627) were selected for monitoring because custom synthesized reference standards became available. However, only one stable-isotope-labeled internal standard, [25,25,25,26,26,26-^2^H_6_]5β-cholestane-3α,7α,12α, 25-tetrol-3-O-β-glucuronide, was available, and this served as the IS for quantification of both bile alcohols. It is not unusual to use a homolog as an internal standard when a stable-isotopically labeled analog is unavailable. A limitation of this approach is that differences in ionization efficiencies can influence the accuracy of the measurement. We assumed similar ionization efficiencies for the bile alcohols tetrol and pentol. This was supported by calibration curves for CTX-611 and CTX-627 that gave similar slopes (0.0009 vs. 0.0008 for CTX-611 and CTX-627, respectively), and this approach yielded consistent and reproducible within-batch and between-batch imprecision (Table 2, Figure S7). Any small inaccuracy in the absolute concentration of the bile alcohol pentol is likely to be clinically irrelevant given the utility of the assay for monitoring responses to therapies.

Mean urinary concentrations of the bile alcohol tetrol and pentol glucuronides in biochemically and/or genetically confirmed CTX were approximately 400- and 800-fold higher, respectively, than the concentrations found in patients with cholestasis or in non-cholestatic controls (Figure 5). The mean concentration of the major urinary bile alcohol glucuronide, 5β-cholestane-3α,7α,12α,23S,25-pentol-glucuronide, in untreated CTX patients was 95.4 ± 12.6 µmol/L (n = 12), and the major tetrol glucuronide, 5β-cholestane-3α,7α,12α,25-tetrol-glucuronide, was 8.2 ± 1.4 µmol/L (n = 21) (Table 3). One-way ANOVA with Type II sums of squares revealed a statistically significant difference in both bile alcohol glucuronides among CTX patients and controls (*p* < 0.001), with a large effect size (ω^2^ > 0.6). Post hoc comparisons using the Games–Howell test showed that CTX patients had significantly higher bile alcohol glucuronides compared to both controls (*p* < 0.001). Both bile alcohol glucuronides were near the detection limit and negligible for cholestatic and non-cholestatic patients, representing clear cutoff values to permit the definitive diagnosis of CTX based on this targeted assay.

#### 3.3.5. Comparison of Targeted LC-MS/MS Assay with Untargeted FAB-MS Analysis

Historically, while GC-MS has been the analytical approach for bile acid analysis, the technique requires time-consuming and manually intensive pre-instrumental steps to extract, purify and derivatize bile acids to increase their thermal stability and volatility. It has not been proven effective for screening large numbers of samples. The secondary ionization technique of FAB-MS is an untargeted technique, meaning new metabolic defects may be detected, and it remains our primary screening approach for diagnosis and therapeutic monitoring of inborn errors of bile acid synthesis, having been in use for over 40 years [1]. It is, however, not considered an acceptable technique for accurate quantification but does provide a semiquantitative assessment of the relative concentrations of the atypical bile acids excreted in urine [1] based on the relative S/N ratio of the ions of the atypical bile acids and the fundamental principle that the intensity of any ion generated in the ion source is proportional to the mass of compound ionized, and thus its concentration [41]. The ability to accurately quantify concentrations of these atypical bile acids is crucial to the evaluation of oral primary bile acid therapy, in which therapeutic efficacy is contingent on suppressing endogenous bile acid synthesis to affect a reduction in the synthesis and urinary excretion of these hepatotoxic metabolites. The targeted tandem mass spectrometry assay described here, interestingly, was found to show a good comparison with the semi-quantitative FAB-MS approach but has the advantage of accurate quantification of atypical bile acids, which is important for evaluating patient response to bile acid therapy [41]. Being a targeted assay, it would not permit the identification of as yet undiscovered bile acid synthesis disorders.

**Figure 6:**
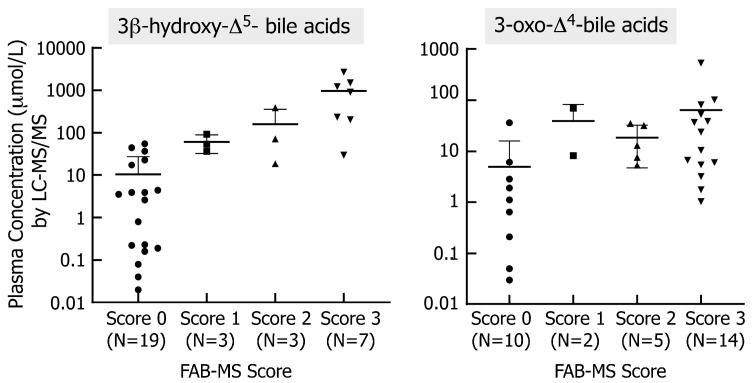
Correlation between the semi-quantitative FAB-MS score and the total urinary 3β-hydroxy-Δ^5^-bile acid and 3-oxo-Δ^4^-bile acid concentrations measured using targeted LC-MS/MS in a subset of randomly selected patients with bile acid synthesis disorders and cholestatic and non-cholestatic controls.

In a blinded manner, we analyzed 63 randomly selected urine samples from patients with HSD3B7 deficiency (n = 21) before and during cholic acid therapy, patients with AKR1D1 deficiency (n = 20), and several cholestatic (n = 7) and healthy controls (n = 5) and compared the concentrations determined using LC-ESI-MS/MS with the previously reported scores assigned to FAB-MS spectra (Figure 4). This FAB-MS semiquantitative method correlated well with the accurate concentrations determined using LC-MS for HSD3B7 deficiency. Samples with an FAB-MS score of 3 had a urinary concentration of total atypical 3-sulfooxy-5-cholen-24- oic acids of 969.3 ± 354.0 µmol/L (n = 7), whereas the concentrations in samples with FAB-MS scores of 0, 1, and 2 were 10.4 ± 3.9 µmol/L (n = 19), 61.4 ± 16.4 µmol/L (n = 3), and 160.0 ± 115.5 (n = 3) µmol/L, respectively. For AKR1D1 deficiency, concentrations in samples with FAB-MS scores of 0, 1, 2 and 3 were 4.9 ± 3.5 µmol/L (n = 10), 38.9 ± 30.7 µmol/L (n = 2), 18.6 ± 6.2 (n = 4) µmol/L, and 64.3 ± 36.8 µmol/L (n = 14), respectively. FAB-MS scores correlated reasonably well with this more accurate LC MS/MS approach (Figure 6) and were in agreement with our previous study [41]. To investigate the correlation, Spearman’s rank correlation coefficients for both HSD3B7 and AKR1D1 deficiencies were computed. The results showed significant positive correlations between the FAB score and LC-MS/MS values. HSD3B7 has a strong correlation (Spearman’s ρ = 0.79, 95% CI [0.62, 0.90], *p* < 0.001), and AKR1D1 showed a moderate but significant correlation (Spearman’s ρ = 0.52, 95% CI [0.20, 0.74], *p* = 0.003). We believe that this result confirms the strength and effectiveness of both approaches.

Furthermore, the LC-MS/MS method has been clinically validated to enable the monitoring of atypical bile acid metabolite concentrations during the treatment of bile acid synthesis disorders (BASDs). This approach overcomes the analytical limitations of previous methods and provides more accurate quantitative, longitudinal data essential for therapeutic monitoring. For instance, oral primary bile acid therapy (e.g., with cholic or chenodeoxycholic acids) suppresses endogenous hepatic bile acid synthesis. This LC-MS/MS method allows clinicians to track the decline in urinary excretion of atypical bile acid metabolites over time, thereby guiding treatment decisions [41].

## 4. Conclusions

A novel assay for the quantification of the key atypical bile acids excreted in the urine of patients with HSD3B7, CYP7B1, CYP27A1 and AKR1D1 deficiencies is described. Normative data for the excretion of specific urinary biomarkers were established for these BASDs, and the enhanced specificity and accuracy of the assay over other less specific or semi-quantitative methods lies in its value to more accurately monitor therapeutic response to primary bile acid therapy.

## Figures and Tables

**Table 1:** List of diagnostically significant MRM transitions (*m*/*z*) monitored for quantification of the atypical metabolites and the stable-isotopically labeled internal standards for the bile acid synthesis disorders of HSB3B7, AKR1D1, CYP7B1 and CYP27A1 deficiency.

Abbreviation	Atypical Bile Acid/Sterol Metabolite	MWt	MRM Transition	Collision Energy (eV)
HSD-469	3β-Sulfooxy-7α-hydroxy-chol-5-en-24-oic	470	469.3 → 96.9	32
HSD-469-IS	[^2^H_5_]3β-Sulfooxy-7α-hydroxy-chol-5-en-24-oic	475	474.3 → 97.9	32
HSD-485	3β-Sulfooxy-7α,12α-dihydroxy-chol-5-en-24-oic	486	485.3 → 96.9	32
HSD-485-IS	[^2^H_5_]3β-Sulfooxy-7α,12α-dihydroxy-chol-5-en-24-oic	491	490.3 → 97.9	32
HSD-526	Glycine conjugate of 3β-sulfooxy-7α-hydroxy-chol-5-en-24-oic	527	262.7 → 96.9	32
HSD-526-IS	[^2^H_2_,^15^N]Glycine conjugate of 3β-sulfooxy-7α-hydroxy- chol-5-en-24-oic	530	265.5 → 96.9	32
HSD-542	Glycine conjugate of 3β-sulfooxy-7α,12α-dihydroxy- chol-5-en-24-oic	543	270.7 → 96.9	32
HSD-542-IS	[^13^C_2_,^2^H_2_,^15^N]Glycine conjugate of 3β-sulfooxy-7α,12α-dihydroxy -chol-5-en-24-oic	548	273.3 → 96.9	32
AKR-444	Glycine conjugate of 3-oxo-7α-hydroxy-chol-4-en-24-oic	445	444.2 → 73.8	40
AKR-444-IS	[^13^C_2_,^2^H_2_,^15^N]Glycine conjugate of 3-oxo-7α-hydroxy- chol-4-en-24-oic	450	449.2 → 78.9	40
AKR-460	Glycine conjugate of 3-oxo-7α,12α-dihydroxy-chol-4-en-24-oic	461	460.2 → 73.8	40
AKR-460-IS	[^13^C_2_,^2^H_2_,^15^N]Glycine conjugate of 3-oxo-7α,12α-dihydroxy- chol-4-en-24-oic	466	465.1 → 78.9	40
AKR-494	Taurine conjugate of 3-oxo-7α-hydroxy-chol-4-en-24-oic	495	494.2 → 342.1	60
AKR-494-IS	[^2^H_4_]Taurine conjugate of 3-oxo-7α-hydroxy-chol-4-en-24-oic	499	498.2 → 346.1	60
AKR-510	Taurine conjugate of 3-oxo-7α,12α-dihydroxy-chol-4-en-24-oic	511	510.2 → 358.0	60
AKR-510-IS	[^2^H_4_]Taurine conjugate of 3-oxo-7α,12α-dihydroxy- chol-4-en-24-oic	515	514.2 → 362.0	60
CYP7-453	3β-Sulfooxy-chol-5-en-24-oic	454	453.3 → 96.9	35
CYP7-453-IS	[2,3,3,23,23-^2^H_5_]3β-Sulfooxy-chol-5-en-24-oic	459	458.3 → 97.9	35
CYP7-510	Glycine conjugate of 3β-sulfooxy-chol-5-en-24-oic	511	510.3 → 96.9	35
CYP7-510-IS	[^13^C_2_,^2^H_2_,^15^N]Glycine conjugate of 3β-sulfooxy-chol-5-en-24-oic	516	515.4 → 96.9	35
CTX-611	5β-Cholestane-3α,7α,12α,25-tetrol-3-O-β-glucuronide	612	611.0 → 84.7	50
CTX-611-IS	[25,25,25,26,26,26-^2^H_6_]5β-Cholestane-3α,7α,12α,25-tetrol- 3-O-β-glucuronide	618	617.0 → 84.7	50
CTX-627	5β-Cholestane-3α,7α,12α,23S,25-pentol-23-O-β-glucuronide	628	626.9 → 84.6	50
CTX-627-IS	Not available	n/a	n/a	n/a

**Table 2:** Within-batch and between-batch imprecision for the major urinary atypical biomarkers of bile acid synthesis disorders, HSD3B7, AKR1D1, CYP7B1 and CYP27A1, determined at three different concentrations (100, 400 and 2000 ng/mL) of quality control samples.

	QC-Low (100 ng/mL) % CV (% Bias)		QC-Med (400 ng/mL) % CV (% Bias)		QC-High (2000 ng/mL) % CV (% Bias)	
	Within-batch	Between-batch	Within-batch	Between-batch	Within-batch	Between-batch
***HSD3B7 deficiency***						
HSD-469	0.6 (9.2)	2.6 (5.6)	0.9 (7.3)	3.0 (1.2)	2.5 (2.8)	2.7 (1.0)
HSD-485	1.2 (10.0)	2.0 (1.1)	0.8 (6.5)	2.5 (0.2)	1.6 (2.3)	1.4 (0.1)
HSD-526	2.4 (11.0)	2.3 (0.1)	2.1 (8.5)	3.6 (−0.1)	1.4 (1.9)	1.6 (0.0)
HSD-542	3.1 (8.5)	3.7 (0.4)	2.8 (9.3)	4.3 (−0.9)	2.4 (2.6)	2.3 (−1.7)
***AKR1D1 deficiency***						
AKR-444	2.2 (9.0)	2.6 (4.6)	1.5 (6.8)	3.1 (2.4)	2.0(2.2)	2.2 (−0.1)
AKR-460	2.3 (8.7)	1.5 (4.9)	2.1 (6.5)	2.0 (3.0)	2.6 (3.9)	1.8 (2.0)
AKR-494	2.2 (5.4)	2.3 (−0.2)	0.9 (6.2)	2.6 (1.0)	2.4 (2.1)	1.8 (1.0)
AKR-510	2.8 (10.0)	3.2 (−0.6)	1.4 (8.1)	2.4 (0.6)	1.1 (3.1)	2.2 (−0.2)
***CYP7B1 deficiency***						
CYP7-453	1.1 (1.7)	2.6 (−3.0)	0.6 (1.4)	2.8 (−3.0)	1.8 (−0.8)	1.4 (−2.3)
CYP7-510	1.6 (4.0)	2.9 (−1.7)	1.3 (1.7)	1.8 (−0.6)	2.0 (−1.5)	2.1 (0.0)
***CYP27A1 deficiency***						
CTX-611	2.6 (−0.4)	6.4 (−1.3)	2.3 (−2.5)	5.5 (1.4)	1.3 (−5.7)	4.2 (6.7)
CTX-627	6.8 (6.9)	6.6 (3.6)	6.8 (8.8)	7.5 (5.8)	9.3 (12.2)	7.8 (5.0)

**Table 3:** Urinary concentrations (mean ± SEM) of the major biomarkers of HSD3B7, AKR1D1 and CYP27A1 deficiencies.

Atypical Metabolite Feature	BASD Mean ± SEM µmol/L (n)	Cholestatic Control Group Mean ± SEM µmol/L (n)	Non-Cholestatic Control Group Mean ± SEM µmol/L (n)	ANOVA (Type II) Analysis		Post Hoc Comparisons (Pair-Wise P)		
				F(df1, df2)	*p*	BASD vs. Chol.	BASD vs. Non-Chol.	Chol. vs. Non-Chol.
***HSD3B7 deficiency***								
HSD-469	68.5 ± 17.2 (22)	0.05 ± 0.01 (168)	0.03 ± 0.01 (127)	110.06(2, 314)	<0.001	0.002	0.002	0.068
HSD-485	66.2 ± 17.1 (22)	0.01 ± 0.00 (168)	0.02 ± 0.02 (127)	103.75(2, 314)	<0.001	0.003	0.003	0.880
HSD-526	291.2 ± 89.0 (22)	0.17 ± 0.04 (168)	0.10 ± 0.02 (127)	74.44(2, 314)	<0.001	0.010	0.010	0.190
HSD-542	278.0 ± 87.1 (22)	0.07 ± 0.02 (168)	0.03 ± 0.01 (127)	74.44(2, 314)	<0.001	0.010	0.010	0.190
Total HSD	703.6 ± 204.2 (22)	0.31 ± 0.04 (168)	0.19 ± 0.05 (127)	82.57(2, 314)	<0.001	0.007	0.007	0.185
***AKR1D1 deficiency***								
AKR-444	9.4 ± 1.4 (48)	0.8 ± 0.1 (168)	0.4 ± 0.3 (127)	79.80(2, 340)	<0.001	<0.001	<0.001	0.470
AKR-460	45.2 ± 12.3 (48)	3.3 ± 0.5 (168)	0.9 ± 0.3 (127)	37.37(2, 340)	<0.001	0.004	0.002	<0.001
AKR-494	6.4 ± 1.2 (48)	0.9 ± 0.1 (168)	0.2 ± 0.1 (127)	65.48(2, 340)	<0.001	<0.001	<0.001	<0.001
AKR-510	20.5 ± 6.5 (48)	4.0 ± 0.4 (168)	0.5 ± 0.3 (127)	23.60(2, 340)	<0.001	0.039	0.010	<0.001
Total AKR	81.4 ± 16.3 (48)	8.9 ± 1.0 (168)	2.0 ± 1.0 (127)	63.35(2, 340)	<0.001	<0.001	<0.001	<0.001
***CYP7B1 deficiency***								
CYP7B-1-453	(2.0, 0.0) (2)	0.04 ± 0.00 (168)	0.01 ± 0.00 (127)	NA *	NA	NA	NA	NA
CYP7B-1-510	(6.1, 8.8) (2)	1.1 ± 0.2 (168)	0.6 ± 0.1 (127)	NA	NA	NA	NA	NA
Total CYP7B1	(8.1, 8.8) (2)	1.1 ± 0.2 (168)	0.6 ± 0.1 (127)	NA	NA	NA	NA	NA
***CYP27A1 deficiency***								
CTX-611	8.2 ± 1.4 (21)	0.02 ± 0.00 (167)	0.02 ± 0.00 (127)	235.51(2, 312)	<0.001	<0.001	<0.001	0.510
CTX-627	95.4 ± 16.7 (12)	0.09 ± 0.01 (76)	0.04 ± 0.00 (30)	153.34(2, 115)	<0.001	<0.001	<0.001	<0.001

Values are mean ± SEM; n denotes number of samples. ANOVA performed using Type II sum of squares. * NA indicates comparison not performed due to insufficient sample size.

## Data Availability

All data generated or analyzed during this study are included in this published article.

## Supplementary Materials

The following supporting information can be downloaded at: https://www.mdpi.com/article/10.3390/metabo16070436/s1. Figure S1. The chromatograms of atypical bile acid metabolites of HSD3B7 deficiency at the limit of low quantification (LLOQ) 50 ng/mL level with signal-to-noise; Figure S2. The chromatograms of atypical bile acid metabolites of AKR1D1 deficiency at the limit of low quantification (LLOQ) 50 ng/mL level with signal-to-noise; Figure S3. The chromatograms of atypical bile acid metabolites of CYP27A1 deficiency at the limit of low quantification (LLOQ) 50 ng/mL level with signal-to-noise; Figure S4. The chromatograms of atypical bile acid metabolites of CYP7B1 deficiency at the limit of low quantification (LLOQ) 50 ng/mL level with signal-to-noise; Figure S5. Receiver operating characteristic (ROC) curve analysis to evaluate the diagnostic performance of urinary atypical bile acid metabolites for distinguishing patients with HSD3B7 deficiency from cholestatic (A) and non-cholestatic (B) controls. Preliminary cutoff value was shown in the figure; Figure S6. Receiver operating characteristic (ROC) curve analysis to evaluate the diagnostic performance of urinary atypical bile acid metabolites for distinguishing patients with AKR1D1 deficiency from cholestatic (A) and non-cholestatic (B) controls. Preliminary cutoff value was shown in the figure; Figure S7. Calibration curves for the CTX-611 and CTX-627 with slopes (0.0009 vs. 0.0008, for CTX-611 and CTX-627 respectively). Table S1. Mass spectrometry parameters used in the LC-MS/MS; Table S2. Recovery (%) of atypical metabolite biomarkers in patients with bile acid synthesis disorders; Table S3. Matrix effects (matrix factor) of atypical metabolite biomarkers in patients with bile acid synthesis disorders.

## Abbreviations

The following abbreviations are used in this manuscript:

BASD	Bile acid synthesis disorders
HSD-469	3β-sulfooxy-7α-hydroxy-chol-5-en-24-oic acid
HSD-485	3β-sulfooxy-7α,12α-dihydroxy-chol-5-en-24-oic acid
HSD-526	Glycine conjugate of 3β-sulfooxy-7α-hydroxy-chol-5-en-24-oic acid
HSD-542	Glycine conjugate of 3β-sulfooxy-7α,12α-dihydroxy-chol-5-en-24-oic acid
HSD-469-IS	[2,3,3,23,23-^2^H_5_]3β-sulfooxy-7α-hydroxy-chol-5-en-24-oic acid
HSD-485-IS	[2,3,3,23,23-^2^H_5_]3β-sulfooxy-7α,12α-dihydroxy-chol-5-en-24-oic acid
HSD-526-IS	[1,2-^13^C_2_, 2,2-^2^H_2_,3-^15^N]glycine conjugate of 3β-sulfooxy-7α-hydroxy-chol-5-en-24-oic acid
HSD-542-IS	[1,2-^13^C_2_, 2,2-^2^H_2_,3-^15^N]glycine conjugate of 3β-sulfooxy-7α,12α-dihydroxy-chol-5-en-24-oic acid
AKR-444	Glycine conjugate of 3-oxo-7α-hydroxy-chol-4-en-24-oic acid
AKR-460	Glycine conjugate of 3-oxo-7α,12α-dihydroxy-chol-4-en-24-oic acid
AKR-494	Taurine conjugate of 3-oxo-7α-hydroxy-chol-4-en-24-oic acid
AKR-510	Taurine conjugate of 3-oxo-7α,12α-dihydroxy-chol-4-en-24-oic acid
AKR-444-IS	[1,2-^13^C2, 2,2-^2^H_2_,3-^15^N]glycine conjugate of 3-oxo-7α-hydroxy-chol-4-en-24-oic acid
AKR-460-IS	[1,2-^13^C_2_, 2,2-^2^H_2_,3-^15^N]glycine conjugate of 3-oxo-7α,12α-dihydroxy-chol-4-en-24-oic acid
AKR-494-IS	[1,1,2,2-^2^H_4_]taurine conjugate of 3-oxo-7α-hydroxy-chol-4-en-24-oic acid
AKR-510-IS	[1,1,2,2-^2^H_4_]taurine of 3-oxo-7α,12α-dihydroxy-chol-4-en-24-oic acid
CYP7-453	3β-sulfooxy-chol-5-en-24-oic acid
CYP7-510	Glycine conjugate of 3β-sulfooxy-chol-5-en-24-oic acid
CYP7-453-IS	[2,3,3,23,23-^2^H_5_]3β-sulfooxy-chol-5-en-24-oic acid
CYP7-510-IS	[1,2-^13^C_2_,2,2-^2^H_2_, 3-^15^N]glycine conjugate of 3β-sulfooxy-chol-5-en-24-oic acid
CTX-611	5β-cholestane-3α,7α,12α, 25-tetrol-3-O-β-glucuronide
CTX-627	5β-cholestane-3α,7α,12α, 23S, 25-pentol-23-O-β-glucuronide
CTX-611-IS	[25,25,25,26,26,26-^2^H_6_]5β-cholestane-3α,7α,12α, 25-tetrol-3-O-β-glucuronide
