# Supplementary material for: Monitoring Atypical Metabolite Biomarkers in Patients with Bile Acid Synthesis Disorders by a Novel Targeted Tandem Mass Spectrometry Assay
Source: Metabolites. 2026 Jun 23;16(7):436. doi: 10.3390/metabo16070436 (PMC13413685; doi:10.3390/metabo16070436)
Supplement: Supplementary file 1 [file metabolites-16-00436-s001.zip › metabolites-4273203-supplementary.pdf]

# Monitoring atypical metabolite biomarkers in patients with bile acid synthesis disorders by a novel targeted tandem mass spectrometry assay

**Kenneth D.R. Setchell[1,2*], Xueheng Zhao[1,2], Stacey Reed[1], and Wujuan Zhang[1,3]**

[1]Clinical Mass Spectrometry, Division of Pathology and Laboratory Medicine, Cincinnati Children's Hospital Medical Center, OH 45229 USA
[2]Department of Pediatrics of the University of Cincinnati College of Medicine, Cincinnati, Ohio 45227, USA
[3]Current address: The CCSG Proteomics and Metabolomics Facility, The Wistar Institute, 3701 Spruce Street, Philadelphia, PA 19104, USA
*Correspondence: Kenneth D.R. Setchell, kenneth.setchell@cchmc.org

**Supplementary captions**

**Figure S1.** The chromatograms of atypical bile acid metabolites of HSD3B7 deficiency at the limit of low quantification (LLOQ) 50 ng/mL level with signal-to-noise.

**Figure S2.** The chromatograms of atypical bile acid metabolites of AKR1D1 deficiency at the limit of low quantification (LLOQ) 50 ng/mL level with signal-to-noise.

**Figure S3.** The chromatograms of atypical bile acid metabolites of CYP27A1 deficiency at the limit of low quantification (LLOQ) 50 ng/mL level with signal-to-noise.

**Figure S4.** The chromatograms of atypical bile acid metabolites of CYP7B1 deficiency at the limit of low quantification (LLOQ) 50 ng/mL level with signal-to-noise.

**Figure S5.** Receiver operating characteristic (ROC) curve analysis to evaluate the diagnostic performance of urinary atypical bile acid metabolites for distinguishing patients with HSD3B7 deficiency from cholestatic (A) and non-cholestatic (B) controls. Preliminary cutoff value was shown in the figure.

**Figure S6.** Receiver operating characteristic (ROC) curve analysis to evaluate the diagnostic performance of urinary atypical bile acid metabolites for distinguishing patients with AKR1D1 deficiency from cholestatic (A) and non-cholestatic (B) controls. Preliminary cutoff value was shown in the figure.

**Figure S7.** Calibration curves for the CTX-611 and CTX-627 with slopes (0.0009 vs 0.0008, for CTX-611 and CTX-627 respectively).

**Table S1.** Mass spectrometry parameters used in the LC-MS/MS

**Table S2.** Recovery (%) of atypical metabolite biomarkers in patients with bile acid synthesis disorders.

**Table S3.** Matrix effects (matrix factor) of atypical metabolite biomarkers in patients with bile acid synthesis disorders.

**Figure S1.**

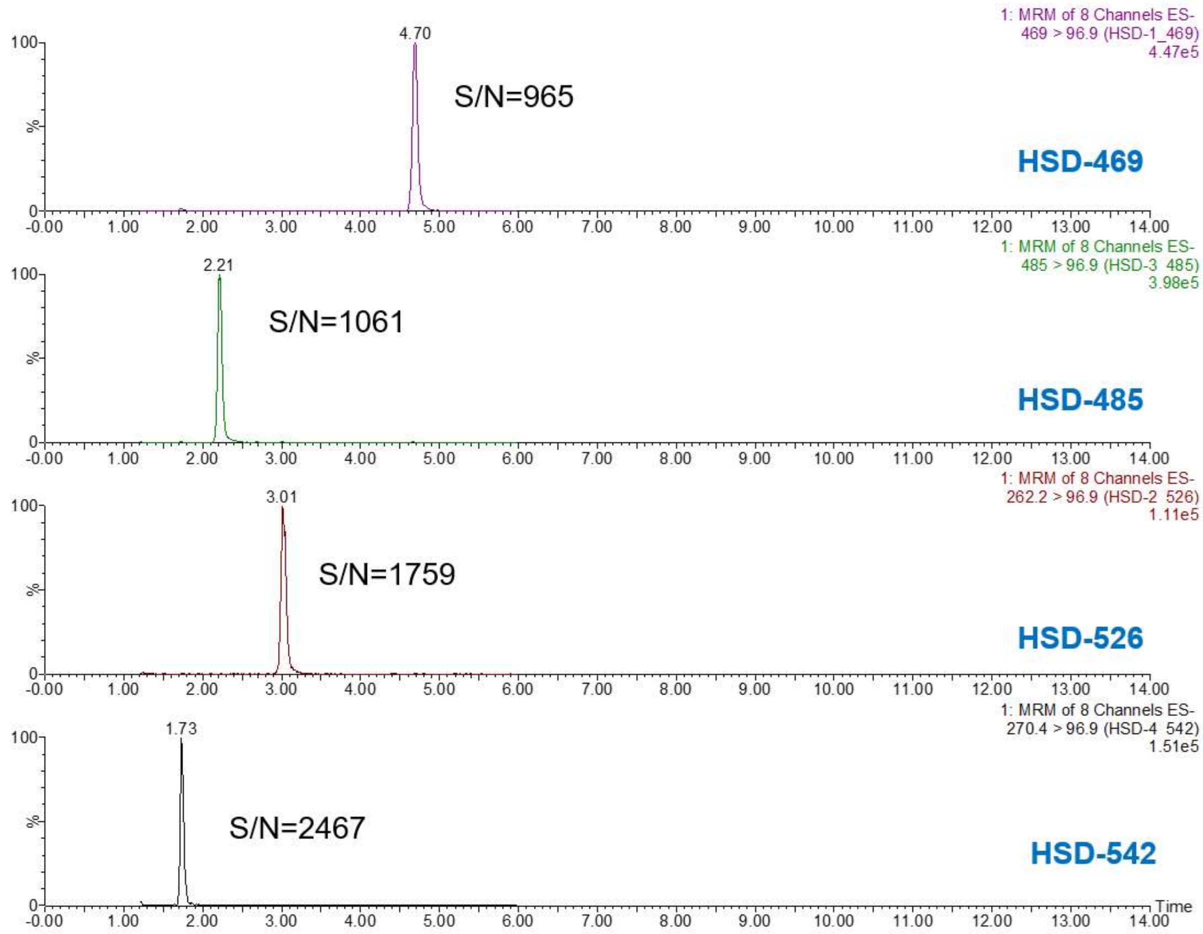
1: MRM of 8 Channels ES-
469 > 96.9 (HSD-1_469)
4.47e5
4.70
S/N=965
HSD-469
1: MRM of 8 Channels ES-
485 > 96.9 (HSD-3 485)
3.98e5
2.21
S/N=1061
HSD-485
1: MRM of 8 Channels ES-
262.2 > 96.9 (HSD-2 526)
1.11e5
3.01
S/N=1759
HSD-526
1: MRM of 8 Channels ES-
270.4 > 96.9 (HSD-4 542)
1.51e5
1.73
S/N=2467
HSD-542
%
Time

**Figure S2.**

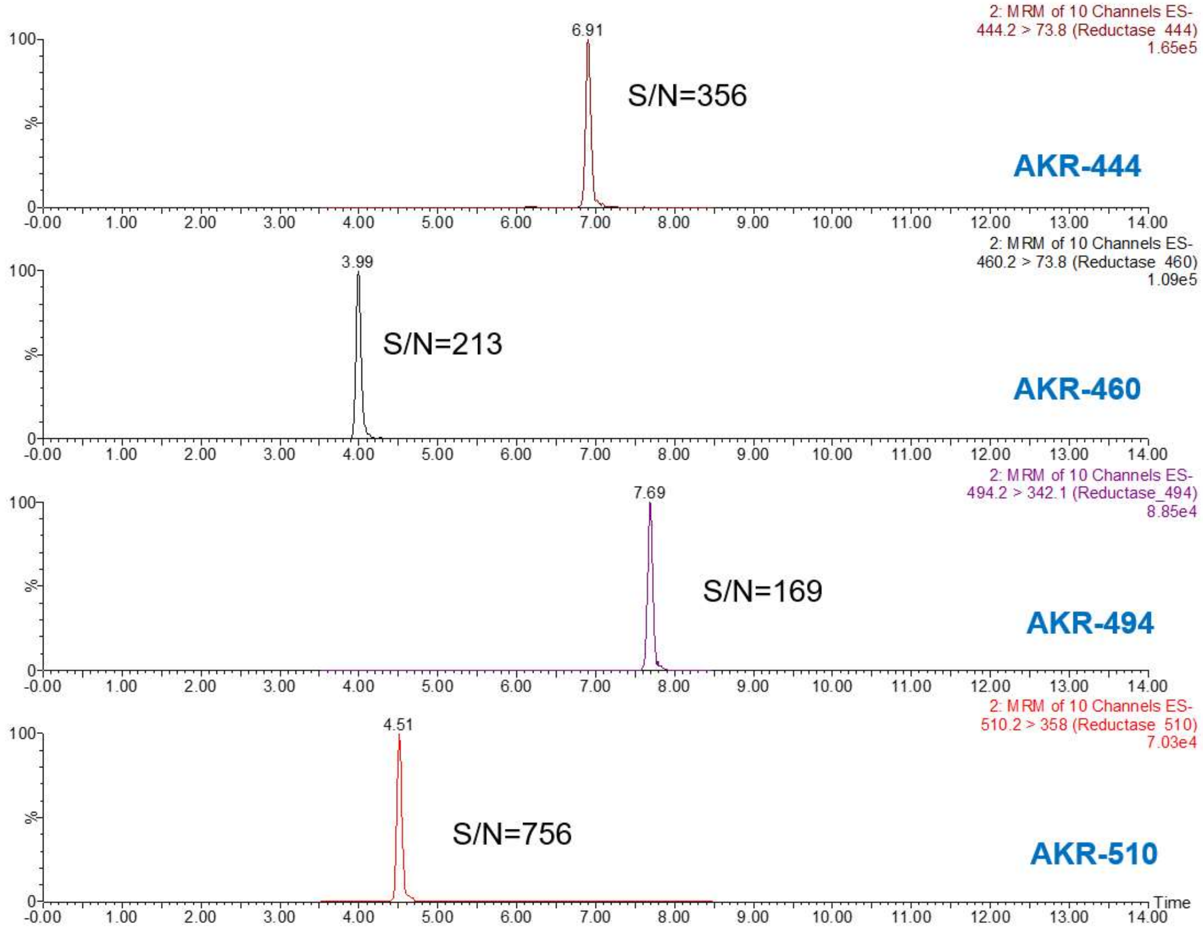
2: MRM of 10 Channels ES-
444.2 > 73.8 (Reductase 444)
1.65e5
6.91
S/N=356
AKR-444
2: MRM of 10 Channels ES-
460.2 > 73.8 (Reductase 460)
1.09e5
3.99
S/N=213
AKR-460
2: MRM of 10 Channels ES-
494.2 > 342.1 (Reductase_494)
8.85e4
7.69
S/N=169
AKR-494
2: MRM of 10 Channels ES-
510.2 > 358 (Reductase 510)
7.03e4
4.51
S/N=756
AKR-510
%
Time

**Figure S3**

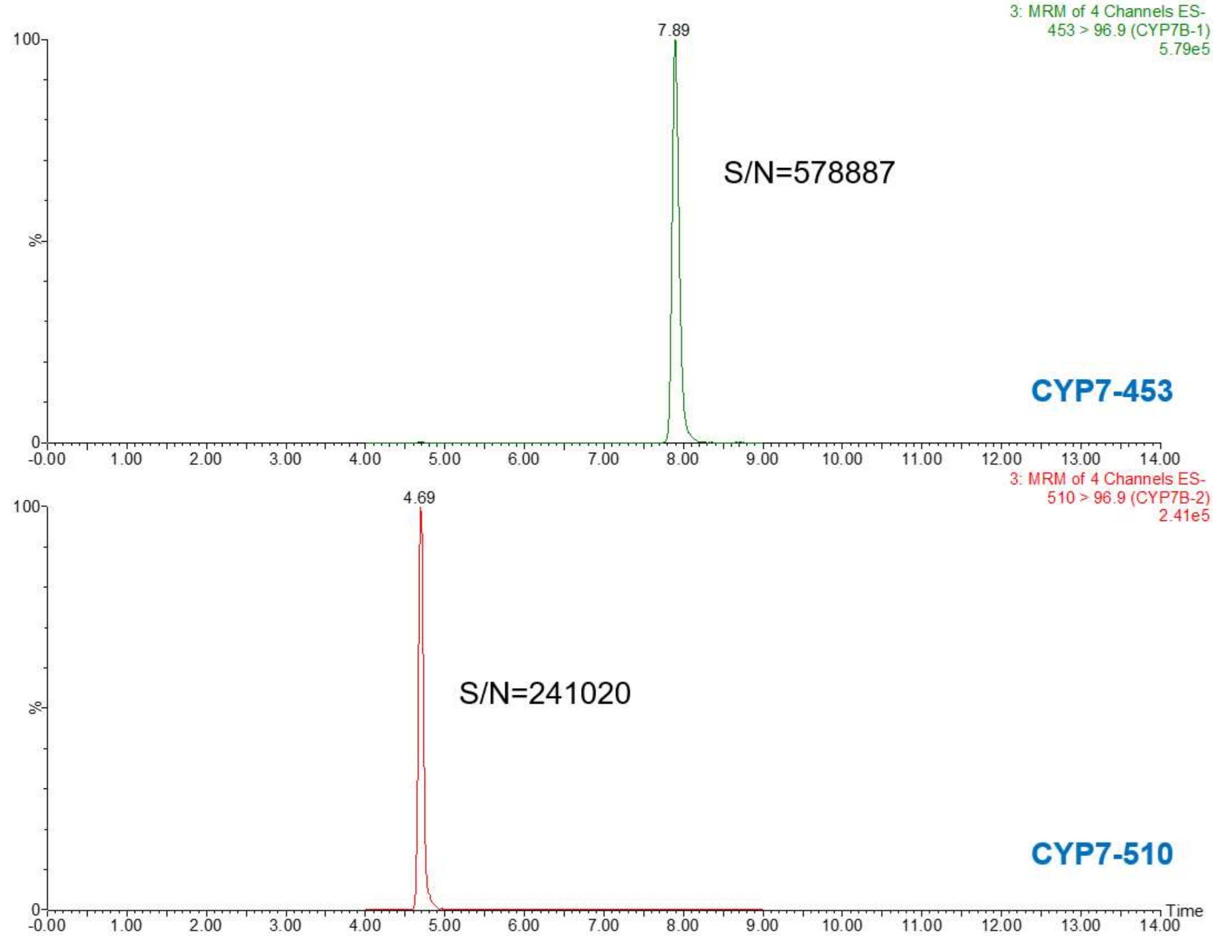
3: MRM of 4 Channels ES-
453 > 96.9 (CYP7B-1)
5.79e5
7.89
S/N=578887
CYP7-453
3: MRM of 4 Channels ES-
510 > 96.9 (CYP7B-2)
2.41e5
4.69
S/N=241020
CYP7-510
Time

**Figure S4**

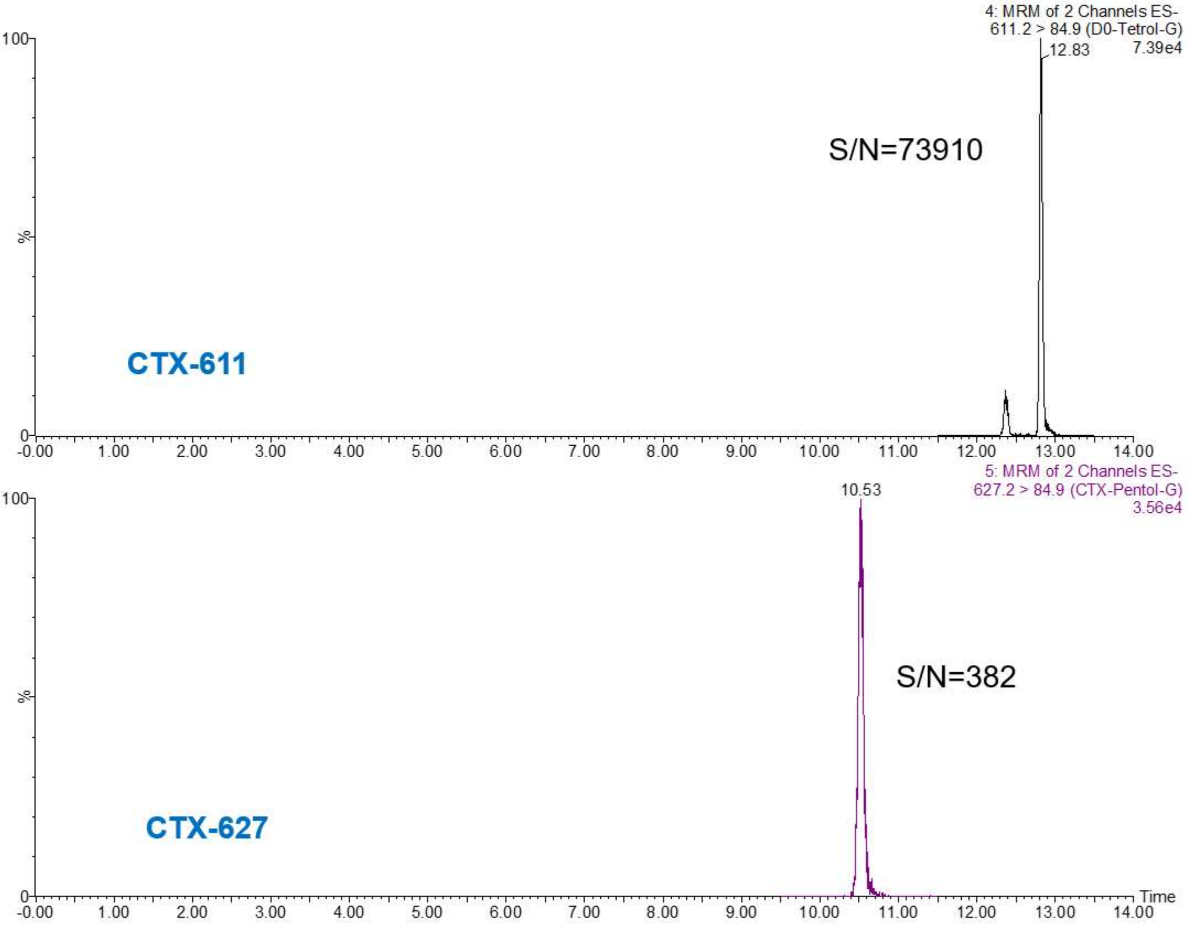
4: MRM of 2 Channels ES-
611.2 > 84.9 (D0-Tetrol-G)
7.39e4
12.83
S/N=73910
CTX-611
10.53
5: MRM of 2 Channels ES-
627.2 > 84.9 (CTX-Pentol-G)
3.56e4
S/N=382
CTX-627
Time
100
%
0
-0.00
1.00
2.00
3.00
4.00
5.00
6.00
7.00
8.00
9.00
10.00
11.00
12.00
13.00
14.00

**Figure S5**

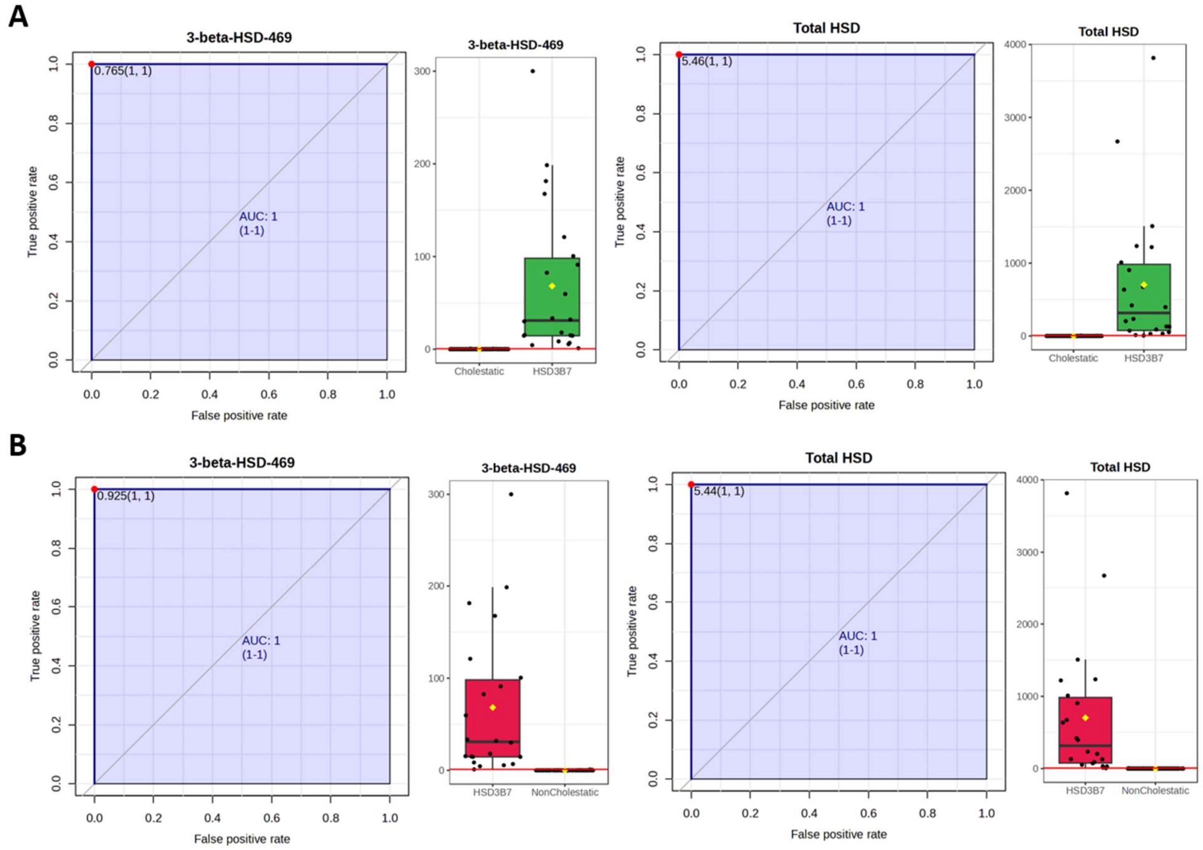
A
3-beta-HSD-469
0.765(1, 1)
AUC: 1
(1-1)
True positive rate
False positive rate
3-beta-HSD-469
Cholestatic
HSD3B7
Total HSD
5.46(1, 1)
AUC: 1
(1-1)
Total HSD
Cholestatic
HSD3B7
B
3-beta-HSD-469
0.925(1, 1)
AUC: 1
(1-1)
3-beta-HSD-469
HSD3B7
NonCholestatic
Total HSD
5.44(1, 1)
AUC: 1
(1-1)
Total HSD
HSD3B7
NonCholestatic

**Figure S6**

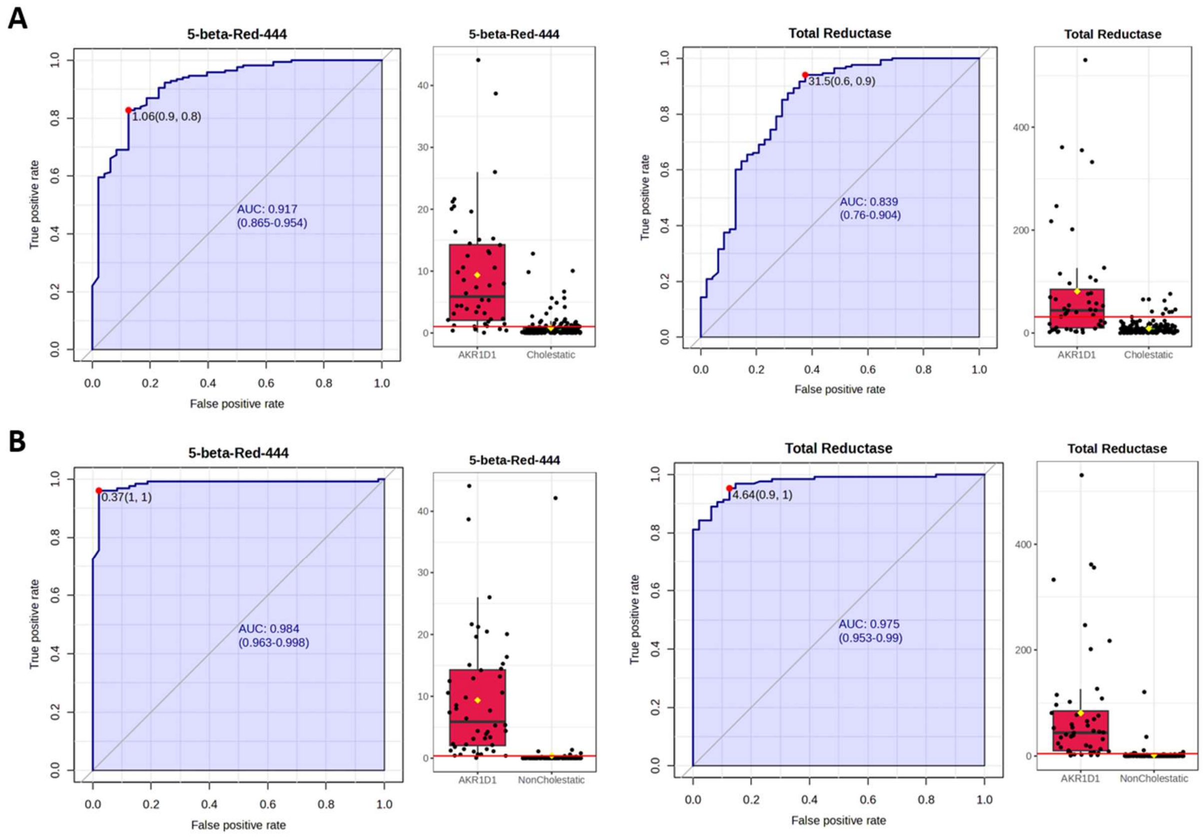
A
5-beta-Red-444
1.06(0.9, 0.8)
AUC: 0.917
(0.865-0.954)
True positive rate
False positive rate
AKR1D1
Cholestatic
Total Reductase
31.5(0.6, 0.9)
AUC: 0.839
(0.76-0.904)
B
5-beta-Red-444
0.37(1, 1)
AUC: 0.984
(0.963-0.998)
AKR1D1
NonCholestatic
Total Reductase
4.64(0.9, 1)
AUC: 0.975
(0.953-0.99)

**Figure S7**

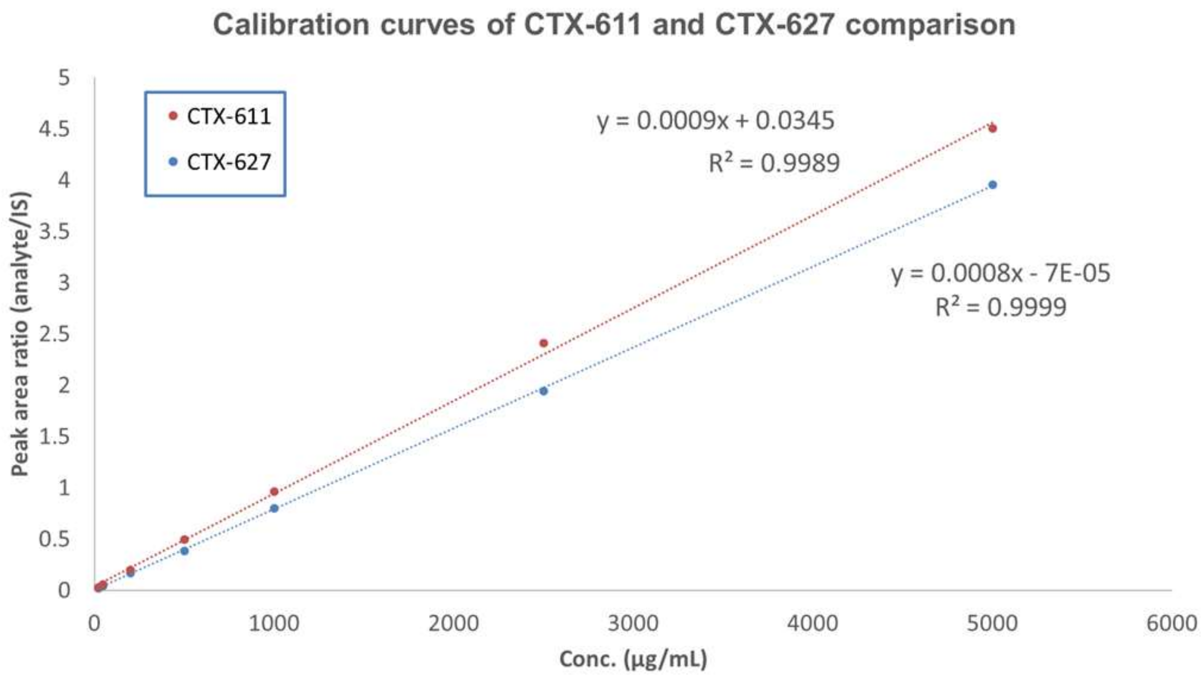

Calibration curves of CTX-611 and CTX-627 comparison
CTX-611
CTX-627
y = 0.0009x + 0.0345
R² = 0.9989
y = 0.0008x - 7E-05
R² = 0.9999
Peak area ratio (analyte/IS)
Conc. (µg/mL)
0
0.5
1
1.5
2
2.5
3
3.5
4
4.5
5
0
1000
2000
3000
4000
5000
6000

**Table S1.** Mass spectrometry parameters used in LC-MS/MS analysis

| MS parameters | |
|---|---|
| Ion Detection Mode | Negative ion |
| Capillary (kV) | 3.0 kV |
| Cone (V) | 30 V |
| Source offset (V) | 30 V |
| Source Temperature (°C) | 150°C |
| Desolvation Temperature (°C) | 500°C |
| Gas Flow – Desolvation (L/h) | 1000 L/h |
| Gas Flow – Cone (L/h) | 150 L/h |
| LM Resolution 1 | 2.6 |
| HM Resolution 1 | 14.5 |
| Ion Energy 1 | 0.5 |
| Entrance | 1 |
| Collision Gas Flow (mL/min) | 0.15 |
| Exit | 1 |
| LM Resolution 2 | 2.8 |
| HM Resolution 2 | 14.7 |
| Ion Energy 2 | 0.5 |
| Collision | Compound dependent, see Table 1 |

**Table S2.** Recovery (%) of atypical metabolite biomarkers in patients with bile acid synthesis disorders.

| Atypical BA | QC-Low (100 ng/mL, n=3) | QC-Med (400 ng/mL, n=3) | QC-High (2000 ng/mL, n=3) | % Recovery (Average) |
|---|---|---|---|---|
| **HSD-469** | 85.7 | 86.5 | 91.6 | 87.9 |
| **HSD-485** | 95.2 | 94.0 | 98.9 | 96.0 |
| **HSD-526** | 92.5 | 96.5 | 105.4 | 98.2 |
| **HSD-542** | 97.8 | 96.6 | 105.2 | 99.9 |
| **AKR-444** | 88.6 | 85.4 | 88.8 | 87.6 |
| **AKR-460** | 90.0 | 87.1 | 93.4 | 90.1 |
| **AKR-494** | 85.4 | 85.6 | 89.9 | 86.9 |
| **AKR-510** | 89.6 | 86.3 | 88.4 | 88.1 |
| **CYP7-453** | 81.0 | 81.6 | 86.7 | 83.1 |
| **CYP7-510** | 79.4 | 77.3 | 81.3 | 79.3 |
| **CTX-611** | 66.4 | 63.8 | 66.7 | 65.6 |
| **CTX-627** | 73.2 | 65.2 | 57.8 | 65.4 |

**Table S3.** Matrix effects (matrix factor) of atypical metabolite biomarkers in patients with bile acid synthesis disorders.

| Atypical BA | QC-Low (100 ng/mL, n=6) | QC-High (2000 ng/mL, n=6) |
|---|---|---|
| **HSD-469** | 0.8 | 0.9 |
| **HSD-485** | 0.9 | 0.9 |
| **HSD-526** | 1.0 | 1.0 |
| **HSD-542** | 1.0 | 1.0 |
| **AKR-444** | 0.9 | 0.9 |
| **AKR-460** | 0.9 | 0.9 |
| **AKR-494** | 0.9 | 0.9 |
| **AKR-510** | 0.9 | 0.8 |
| **CYP7-453** | 1.0 | 0.9 |
| **CYP7-510** | 0.7 | 0.8 |
| **CTX-611** | 0.9 | 0.9 |
| **CTX-627** | 1.0 | 1.0 |
